# Potential Involvement of Oxidative Stress, Apoptosis and Proinflammation in Ipconazole-Induced Cytotoxicity in Human Endothelial-like Cells

**DOI:** 10.3390/toxics11100839

**Published:** 2023-10-05

**Authors:** Iris Ruiz-Yance, Junior Siguas, Brandy Bardales, Ingrid Robles-Castañeda, Karen Cordova, Alina Ypushima, Esteban Estela-Villar, Carlos Quintana-Criollo, Darwin Estacio, José-Luis Rodríguez

**Affiliations:** 1 Agroforestry Department, Universidad Nacional Intercultural de la Amazonia, Pucallpa 25004, Peru; iruizy@unia.edu.pe (I.R.-Y.); bbardalesc@unia.edu.pe (B.B.); iroblesc@unia.edu.pe (I.R.-C.);; 2 Animal Physiology Department, Universidad Nacional Mayor de San Marcos, Lima 15021, Peru; 3 Pharmacology and Toxicology Department, Universidad Complutense de Madrid, 28040 Madrid, Spain

**Keywords:** cytotoxicity, EA.hy926, ipconazole fungicide, oxidative stress, proinflammation

## Abstract

Triazole fungicides are widely used in the world, mainly in agriculture, but their abuse and possible toxic effects are being reported in some in vivo and in vitro studies that have demonstrated their danger to human health. This in vitro study evaluated the cytotoxicity, oxidative stress and proinflammation of EA.hy926 endothelial cells in response to ipconazole exposure. Using the MTT assay, ipconazole was found to produce a dose-dependent reduction (*** *p* < 0.001; concentrations of 20, 50 and 100 µM) of cell viability in EA.hy926 with an IC_50_ of 29 µM. Also, ipconazole induced a significant increase in ROS generation (** *p* < 0.01), caspase 3/7 (** *p* < 0.01), cell death (*BAX*, *APAF1*, *BNIP3*, *CASP3* and *AKT1*) and proinflammatory (*NLRP3*, *CASP1*, *IL1β*, *NFκB*, *IL6* and *TNFα*) biomarkers, as well as a reduction in antioxidant (*NRF2* and *GPx*) biomarkers. These results demonstrated that oxidative stress, proinflammatory activity and cell death could be responsible for the cytotoxic effect produced by the fungicide ipconazole, such that this triazole compound should be considered as a possible risk factor in the development of alterations in cellular homeostasis.

## 1. Introduction

**Figure 1:**
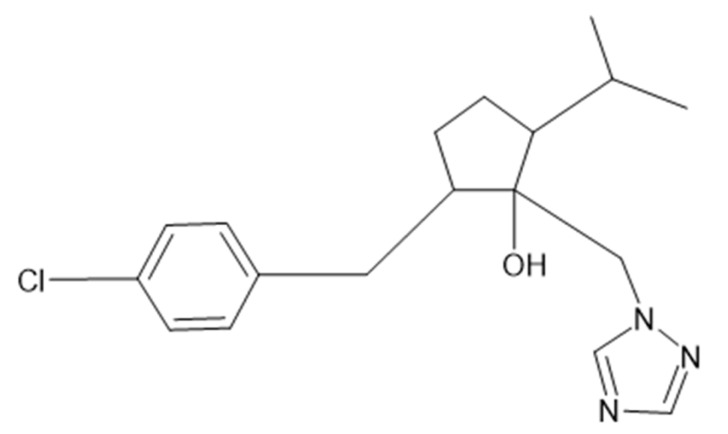
Chemical structure of ipconazole: 2-[(4-chlorophenyl)methyl]-5-propan-2-yl-1-(1,2,4-triazol-1-ylmethyl)cyclopentan-1-ol.

Ipconazole is a broad-spectrum fungicide used as an agrochemical and pesticide to protect plants of various crops, turfs, ornamental flowers and conifers. Ipconazole is structurally similar to other triazole compounds used as pesticides (Figure 1), and its mechanism of action is to inhibit sterol synthesis in fungi, especially zygomycetes, ascomycetes, basidiomycetes and deuteromycetes, at concentrations of 20 g/ton of seed. There are few studies on the tolerance to the residues of concern by ipconazole in plant and animal products. However, some toxicological information is available such as the LD50 in rats for acute oral (1338 mg/kg males and 888 mg/kg females), acute dermal (>2000 mg/kg both sexes) and acute respiratory (1.88 mg/L both sexes) toxicity. In terms of chronic toxicity/carcinogenicity, ipconazole belongs to the triazole class, and several chemicals in this class induced liver tumors in mice [1,2].

Triazole fungicides such as ipconazole are widely used worldwide as agrochemical pesticides; as with all pesticides, only small quantities act on the targets, and most of the remaining residues become environmental contaminants that enter agricultural soils, water or air [3]. In addition, several fungicide formulations may be less degradable and more persistent in the environment, which aggravates contamination, leading to the increased exposure of non-target organisms such as humans and animals [4,5]. Several forms of exposure to triazole fungicides such as dietary exposure via contaminated food, non-food exposure via dermal contact and inhalation of these pesticides in contaminated sites, which pose a risk to human health, were also reported [6]. Other experimental studies showed that triazoles can induce cytotoxicity in different animal cells and tissues, which includes affecting reproduction and development in mammals [7,8,9,10].

The cytotoxic effects produced by triazole fungicides may mainly be associated with oxidative stress mediated by reactive oxygen species (ROS), excessive levels of which can cause damage to cellular components, even leading to cell death [11]. Many in vitro and in vivo studies already showed that pesticides of various types (pyrethroids, organophosphates, chlorinated, etc.) can produce toxicological effects related to high ROS production in cell lines and animal tissues; these oxidative effects are also described as one of the main mechanisms of action of triazole fungicides [12,13]. It was reported that triazoles produce significant increases in ROS in mammalian cell lines PC12 and HepG2 [14,15]. Other studies found high ROS production in the liver and kidney tissue of rats exposed to triazoles [16,17]. In addition, ROS can induce cellular responses such as the activation of the inflammasome complex [18] or apoptosis [19]. The inflammasome is a cytosolic protein complex that, when activated, can induce a strong inflammatory response, triggering the release of proinflammatory cytokines interleukin 1β and interleukin 18 (IL-18) and even inducing cell death [20]. In contrast, the function of apoptosis is to eliminate dysfunctional cells, and the BCL2 family is the main mediator of apoptosis. Bax and Bak, two proapoptotic molecules belonging to the BCL2 family that, when activated, allow for the permeability of the mitochondrial membrane [21] and the release of several proteins such as Cyt c, which trigger the activation of caspases enzymes and, ultimately, apoptosis [22].

Considering that more information is needed on the cytotoxic effects of triazole fungicides, the present study evaluated the cytotoxicity of the fungicide ipconazole in a human endothelial EA.hy926 model. The toxic effects of ipconazole were determined by a cell viability assay (MTT), intracellular ROS generation and caspase 3/7 apoptotic enzyme activity, and these assays were used to determine the working concentrations of ipconazole for the assessment of the molecular expression of genes related to cell death, inflammasome complex activation and antioxidant biomarkers.

## 2. Materials and Methods

### 2.1. Reagents

Ipconazole (C_18_H_24_ClN_3_O) with a purity of 98.96% (g/g) was obtained from LGC group (Barcelona, Spain). Thiazolyl blue tetrazolium bromide (MTT), gentamicin, penicillin G, streptomycin, 2′,7′-Dichlorofluorescein diacetate (DCFH-DA) and Dulbecco’s phosphate buffered saline were obtained from Sigma-Aldrich (Madrid, Spain), Dimethyl sulfoxide (DMSO) and all other usual laboratory reagents were acquired from Panreac (Barcelona, Spain). Kits for RNA extraction, retrotranscription and real-time PCR, DMEM culture media and fetal bovine serum (FBS) were from Cultek (Madrid, Spain). The Apo-ONE Homogeneous Caspase-3/7 Assay kit was acquired from Promega (Madison, WI, USA).

### 2.2. Cell Culture

Human EA.hy926 cells were cultured and passaged in DMEM-FBS and antibiotics. Cells were incubated in humidity with 5% CO_2_ and 95% air and at 37 °C. Cells were treated with ipconazole (1, 5, 10, 20, 50 and 100 µM, dissolved in DMSO) for 24 h. A vehicle group (0.1% DMSO) was used in each experiment. Cells were used with less than 15 passages [18].

### 2.3. Cell Viability Evaluation (MTT)

MTT assay was based on the reduction of the tetrazolium salt (yellow) to formazan (purple) due to the action of active mitochondria [19]. After ipconazole treatments, 0.5 mg/mL MTT was added to each well and incubated for 2 h to allow mitochondrial reduction of the tetrazolium. After 2 h of incubation, the supernatant was removed from each well, and 150 µL of DMSO was added. The absorbance was measured at 540 nm (SPECTROstar BMG microplate reader). Cell viability is represented as percentage of control.

### 2.4. ROS Production

Briefly, after ipconazole treatments, 10 µM of DCFH-DA was added to each well (2 × 10^5^ cells/well under incubation conditions) of a black multi-well plate, incubated for 30 min and quantified in a fluorescent microplate reader (FLx800 fluorimeter, BioTek, VT, USA) at 485 nm/530 nm (λ excitation/λ emission) [18].

### 2.5. Apoptotic Assay with Caspase 3/7 Activity

EA.hy926 cells (15 × 10^3^ cells/well) were seeded in black 96-well plates. After ipconazole treatments, Apo-ONE Caspase 3/7 was prepared and used according to the manufacturer’s instructions. The buffer lyses cultured EA.hy926 cells, and the substrate and buffer were combined to make the Apo-ONE Caspase-3/7 Reagent that was directly added to each well. The fluorescence at 485/528 nm (λ excitation/λ emission) was measured with the plate reader (FLx800, BioTek, Winooski, VT, USA). Data are presented as percentage of control [18].

### 2.6. Molecular Biomarkers Assessment

EA.hy926 cells were seeded in 25 mL flasks in triplicate for each treatment: control, vehicle and ipconazole (20 µM and 50 µM). For the molecular assays, we decided to use 2 concentrations of ipconazole producing a possible mild effect (20 µM) and a possible marked effect (50 µM), which were selected with reference to the IC_50_ (29 µM). After 24 h of treatments, total RNA was obtained according to the manufacturer’s conditions. Total RNA was quantified using a nano spectrophotometer (A260/A280 ratios close to 2.0 in all samples). After the cDNA synthesis by retro-transcription, real-time PCR assays were performed to assess the expression of genes related to cell death, inflammasome complex and antioxidant capacity with a real-time PCR system. For RT-PCR, 400 nM of primers were used (Table 1), and the thermocycling protocol was 95 °C for 2 min and 40 cycles of 5 s at 95 °C and of 30 s at 60 °C. The efficiency of the raw data was analyzed using LinRegPCR software 20210614 [18].

### 2.7. Statistics

To determine whether the data followed the normal distribution, the non-parametric Shapiro–Wilk test (*n* < 50) was used with a significant value of *p* ≤ 0.05. All data analyzed for cell viability, ROS production, caspase 3/7 activity, cell death/inflammasome complex and antioxidant biomarkers were found to be non-significant (*p* > 0.05) by the Shapiro–Wilk test, confirming the normality of the data, and a parametric test was used to determine significance among the groups studied. Statistical analysis of the data was performed by one-way ANOVA and Dunnett’s post hoc with a confidence level of *p* < 0.05, using GraphPad Prism statistical software version 8.0. IC_50_ value was calculated by concentration–response (sigmoidal fitting) with OriginPro 8.0 software.

## 3. Results

### 3.1. Cell Viability

**Figure 2:**
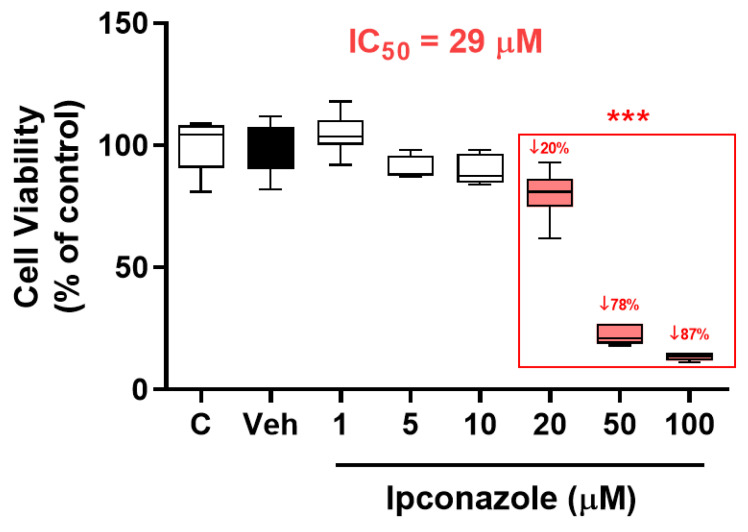
Reduction in cell viability produced by the fungicide ipconazole. Concentrations of 20, 50 and 100 µM ipconazole produced a significant, dose-dependent reduction in cell viability of EA.hy926 after a 24 h exposure period. Data are presented as percentage of control and as mean ± SD of six replicates. Concentrations of ipconazole (red boxes) show significant differences (*** *p* < 0.001) compared to vehicle (Veh; black box).

The MTT assay was used to assess cell viability. EA.hy926 endothelial cells were exposed to ipconazole fungicide (1, 5, 10, 20, 50 and 100 µM) for 24 h, showing a significant (*** *p* < 0.001) reducing effect on cell viability in a dose-dependent manner (Figure 2). The results showed that cell viability was reduced by 20%, 78% and 87% by the effect of ipconazole at concentrations of 20, 50 and 100 µM, respectively, compared to vehicle. The IC_50_ value was also determined to be 29 µM.

### 3.2. ROS Generation

**Figure 3:**
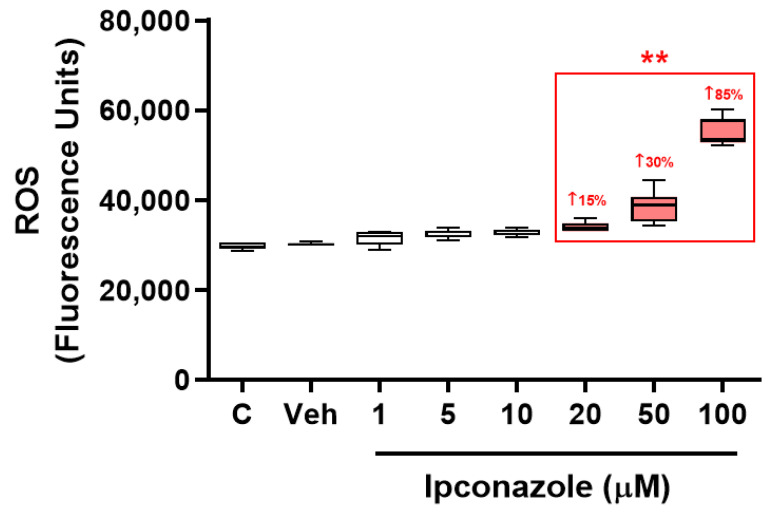
ROS generation induced by the fungicide ipconazole. Concentrations of 20, 50 and 100 µM ipconazole significantly increased ROS generation in EA.hy926 cells after a 24 h exposure period. Data are presented as percentage of control and mean ± SD of six replicates. Concentrations of ipconazole (red boxes) show significant differences (** *p* < 0.01) compared to vehicle (Veh; black box).

EA.hy926 endothelial cells were exposed to the fungicide ipconazole for 24 h to assess whether this compound is able to generate oxidative alterations, which was measured by the intracellular ROS generation. It was observed that ipconazole significantly (** *p* < 0.01) increased ROS generation at concentrations of 20 (15%), 50 (30%) and 100 (85%) µM, reflecting that it acted as a pro-oxidant substance (Figure 3).

### 3.3. Caspase 3/7 Activity

**Figure 4:**
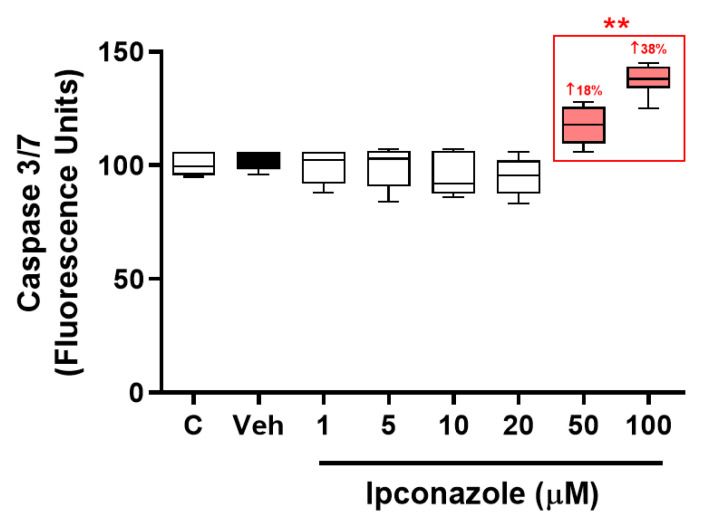
Enzymatic activity of caspase 3/7 by the fungicide ipconazole. Concentrations of 50 and 100 µM ipconazole significantly increased caspase 3/7 enzymatic activity in EA.hy926 cells after a 24 h exposure period. Data are presented as percentage of control and mean ± SD of six replicates. Concentrations of ipconazole (red boxes) show significant differences (** *p* < 0.01) compared to vehicle (Veh; black box).

Cell death is an intracellular event involving the activation of several proapoptotic molecules such as caspases. In the present study, caspase 3/7 enzyme activity was evaluated as an indicator of possible cell death in EA.hy926 cells exposed to the fungicide ipconazole. It was observed that ipconazole was able to significantly (** *p* < 0.01) induce caspase 3/7 enzyme activity at concentrations of 50 and 100 µM by 18% and 38%, respectively (Figure 4).

After observing that cell viability and ROS generation were altered at 20 µM ipconazole, that caspase 3/7 increased at 50 µM and that the IC_50_ was 29 µM, it was decided to use two concentrations of ipconazole, one below (20 µM) and one above (50 µM) the IC_50_ for the molecular assays.

### 3.4. Biomarkers of Cell Death

**Figure 5:**
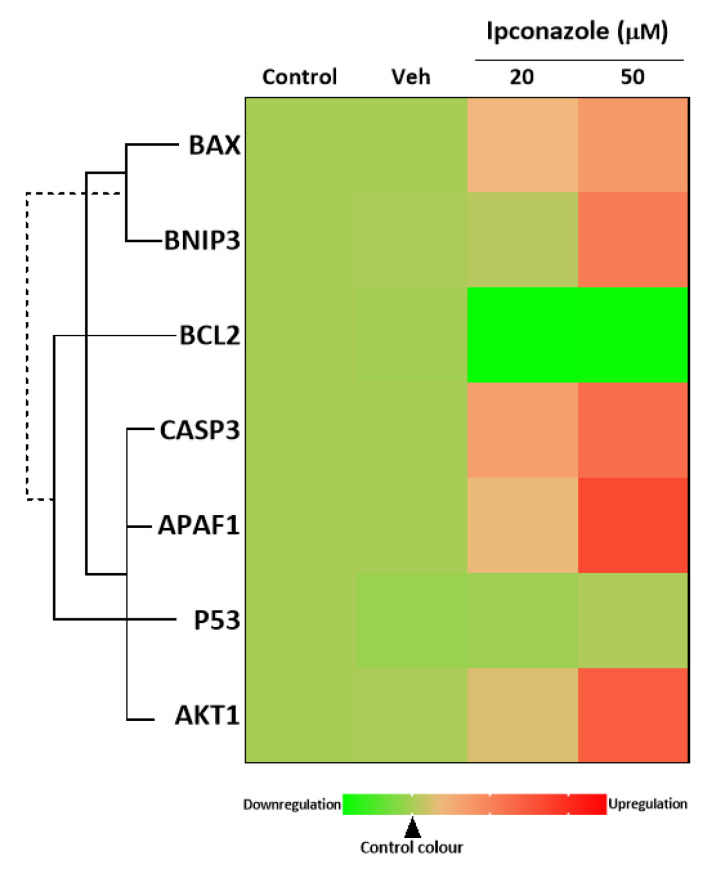
Heat map of the association between cell-death-related biomarkers (BAX, BNIP3, BCL2, APAF1, P53 and AKT1) in EA.hy926 cells after 24 h of exposure to ipconazole (20 and 50 µM). The green color scale represents down-regulation, and the red color scale represents up-regulation, relative to vehicle (0.1% DMSO). The solid line is a direct association, and the dashed line is an inverse association.

Using qPCR molecular analysis, we assessed whether the molecular expression of cell death biomarkers was altered by ipconazole in EA.hy926 cells. Ipconazole at concentrations of 20 and 50 µM resulted in a significant increase in the molecular expression of *BAX* (1.39- and 1.73-fold, respectively) and *CASP3* (1.66- and 2.24-fold, respectively). In addition, only the highest concentration of ipconazole (50 µM) increased the molecular expression of *APAF1* (2.66-fold), *BNIP3* (2.08-fold) and *AKT1* (2.42-fold), compared to vehicle (Table 2). However, ipconazole at concentrations of 20 and 50 µM down-regulated *BCL2* gene expression by 0.13-fold and 0.16-fold, respectively. These data suggested a possible apoptotic effect of ipconazole exposure on EA.hy926 endothelial cells (Table 2). Figure 5 shows the heat map of the direct or inverse association between cell-death-related genes that were up- or down-regulated by ipconazole.

### 3.5. Inflammasome Complex

**Figure 6:**
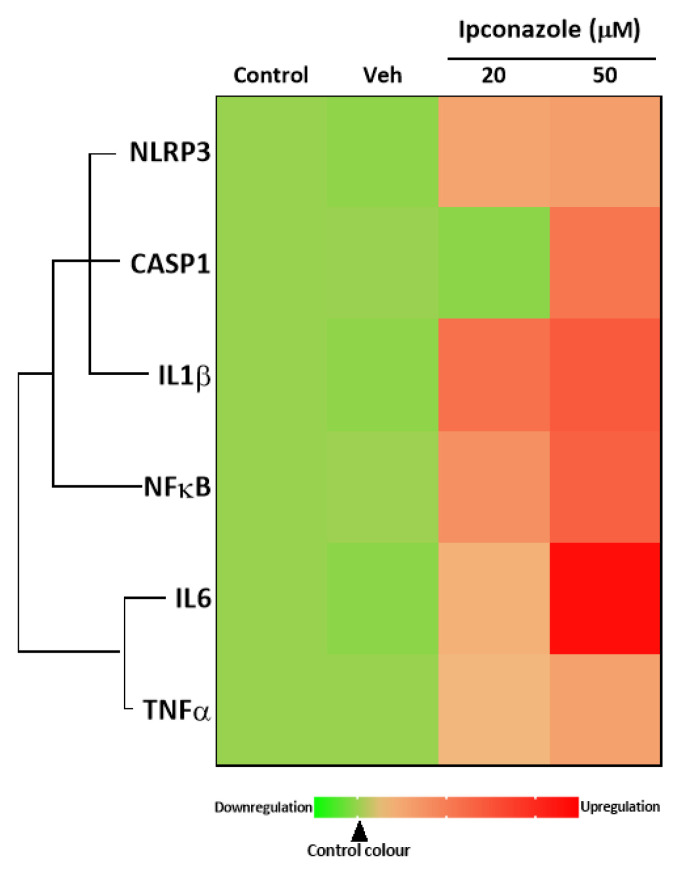
Heat map of the association between inflammasome-related biomarkers (NLRP3, CASP1, IL1β, NFκB, IL6 and TNFα) in EA.hy926 cells after 24 h of exposure to ipconazole (20 and 50 µM). The green color scale represents down-regulation, and the red color scale represents up-regulation, relative to vehicle (0.1% DMSO). The solid line is a direct association.

In this study, the gene expression of molecules involved in the activation, formation and response of the inflammasome complex was assessed by qPCR. In EA.hy926 endothelial cells exposed to ipconazole for 24 h, it was observed that at concentrations of 20 and 50 µM it produced a significant increase in the molecular expression of IL1β by 2.14- and 2.41-fold, respectively, compared to vehicle. Furthermore, only the highest concentration of ipconazole (50 µM) produced an increase in the molecular expression of NLRP3 (1.59-fold), CASP1 (2.07-fold), NFκB (2.31-fold), IL6 (3.34-fold) and TNFα (1.56-fold), compared to vehicle (Table 3). Figure 6 shows the heat map of the direct associations among the inflammasome-complex-related genes that were up-regulated by ipconazole.

### 3.6. Antioxidant Molecular Biomarkers

**Figure 7:**
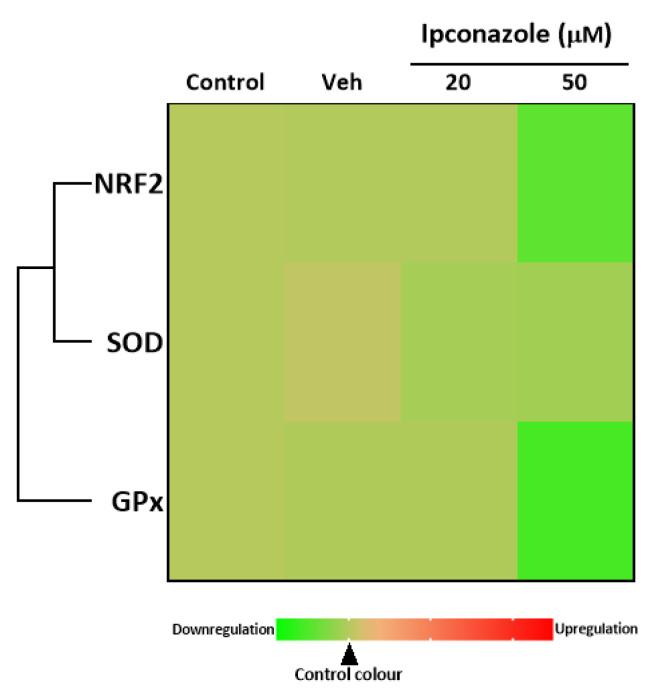
Heat map of the association between antioxidant biomarkers (*NRF2*, *SOD* and *GPx*) in EA.hy926 cells after 24 h of exposure to ipconazole (20 and 50 µM). The green color scale represents down-regulation, and the red color scale represents up-regulation, relative to vehicle (0.1% DMSO). The solid line is a direct association.

The importance of studying the molecular expression of antioxidants lies in the fact that these molecules can maintain or restore homeostasis, which may be altered because of a cellular challenge. In the present study, it was observed that ipconazole (50 µM) produced a significant decrease in *NRF2* (0.56-fold) and *GPx* (0.45-fold) expression, compared to the vehicle (Table 4). Figure 7 shows the heat map of the direct associations among the antioxidant-related genes that were up-regulated by ipconazole. These data may indicate that elevated ROS production or other mechanisms involved may be attenuating or depleting the cellular antioxidant pathway.

## 4. Discussion

The pesticide ipconazole is one of the most widely used triazole fungicides in agriculture to protect crops from various fungal diseases. The abuse of fungicides such as ipconazole is becoming a problem for human, animal and plant health due to its widespread use in various agricultural products [2].

The present study evaluated the cytotoxic effects of the fungicide ipconazole at different concentrations (1–100 µM) on the endothelial cell line EA.hy926, showing that ipconazole (IC_50_ = 29 µM) decreased the cell viability of EA. hy926 cells in a dose-dependent manner; this cytotoxic effect was also observed for other fungicides in various cell cultures [23,24,25,26,27,28], as in the case of tebuconazole (IC_50_ = 60 µM), which produced cytotoxicity in cardiomyocytes and H9c2 cells [10]. And, when tebuconazole was used at concentrations of 0–160 µM, it also induced dose-dependent cytotoxicity in HepG2 and HepaRG cells [23]. In other studies, epoxiconazole (10–60 µM) was used to assess the cytotoxicity it could generate in rat neuronal cells and was shown to significantly inhibit cell viability after 24 h of treatment [24]; when epoxiconazole (IC_50_ = 50 µM) was exposed to F98 cells, cell viability was decreased, and cell morphology was altered [25]; and, in bovine lymphocytes, epoxiconazole was shown to slightly decrease cell viability, with up to 70% of cells undergoing apoptosis at the highest concentration (15 µg mL^−1^) [26]. In SH-SY5Y nerve cells exposed to propiconazole for 4 h, a significant reduction in cell viability was observed relative to the control at 200 µM, and, at 24 h and 48 h, there was a significant reduction in cell viability at concentrations of 100 and 200 µM [9]; while in Hepa1c1c7 and HepG2 cell lines exposed to propiconazole (0–400 µM) for 24 h, 50% viability was observed for Hepa1c1c7 cells at 85.4 µM, and for HepG2 cells it was 148.4 µM [27]. In the murine neural embryonic stem cell assay, cyproconazole affected cell viability in a concentration-dependent manner, producing an IC_20_ of 117.6 µM [28]. The present study and those mentioned in this section support the cytotoxic activity of triazole fungicides as potential environmental contaminants to be considered as a factor in the development of chronic diseases in humans.

Numerous conditions intrinsic or extrinsic to cellular activity, such as exposure to pesticidal pollutants, affect cellular stability by inducing a predominantly ROS-governed oxidative response, which promotes the cellular damage of biomolecules such as proteins, lipids and nucleic acids [18,19,29]. In contrast, to avoid the deleterious effects that excess ROS production can cause, antioxidant defense generated by mediators (NRF2) and antioxidant enzymes (*SOD*, *GPx* and others) is an efficient response to a cellular oxidative imbalance [30,31,32]. NRF2 is a transcription factor that regulates cellular antioxidant action by stimulating the mRNA transcription of antioxidant enzymes that reduce ROS production. The enzyme *SOD* accelerates the reaction of superoxide anion (O2^•−^) to form hydrogen peroxide and oxygen (2O_2_^•−^ + 2H^+^→H_2_O_2_ + O_2_), controlling O2^•−^; it also controls the formation of ROS [31]. Whereas *GPx* allows water formation from H_2_O_2_, hence it is important to find both antioxidant enzymes in subcellular compartments to maintain oxidative homeostasis [32]. In the present study, we evaluated the effect of ipconazole on ROS generation by fluorescence and the mRNA molecular expression of the antioxidant biomarkers *NRF2*, *SOD* and *GPx* in the EA.hy926 cell line. Our results showed that ipconazole above 20 µM induced ROS generation in a dose-dependent manner, while *NRF2* and *GPx* mRNA expression was down-regulated. Data from other studies linking oxidative stress by fungicidal effects showed that epoxiconazole significantly induced ROS generation in PC12 cells at concentrations of 5, 10 and 20 µM [33]; in neural stem cells at concentrations of 2, 5 and 10 µM [24]; in F98 nerve cells at a concentration of 50 µM [34]; and in HCT116 cells at concentrations of 10, 20 and 30 µM [35]; that penconazole at 1 and 2 mg/L significantly increased ROS production in zebrafish [36]; that tebuconazole induced a significant increase in O2^•−^ in H9c2 cells at concentrations of 30 and 60 µM [10] and in human colon carcinoma HCT116 cells at 25 and 50 µM [37]; that propiconazole increased ROS levels in AML12 mouse liver hepatocytes from 25 to 100 µM [38]; that flusilazole and imazalil induced ROS production, although only after exposure to 100 µM in PC12 cells [14]; and that cyproconazole, flusilazole, hexaconazole, prochloraz and tebuconazole at 10 µM produced significant increases in ROS in murine Leydig cells (MA-10) and human T47D-ARE cells [39]. These in vitro data demonstrated that ROS generation can be induced in various cell types by triazole fungicides.

Briefly, the main response to oxidative stress is the activity of antioxidant molecules such as *NRF2*, *SOD*, *GPx* and others. In this study, antioxidant mediators (*NRF2* and *GPx*) were down-regulated in EA.hy926 cells by ipconazole (20 and 50 µM). A similar response was also observed with epoxiconazole (56 mg/kg), which reduced the activities of the antioxidant enzymes catalase, GPx and glutathione transferase in rat kidney and liver cells [25]. However, other studies indicated that triazole fungicides are able to increase the antioxidant response after an oxidative challenge [34,40].

Cell death, commonly known as apoptosis, is a highly regulated and controlled cellular process in which cells with their physiology affected by an exogenous agent or by a product of their metabolism are eliminated from a tissue. Apoptosis is mediated by initiator and effector caspases that induce the various processes involved in cell destruction. These processes include DNA fragmentation by DNAase, chromatin condensation and membrane rupture by actomyosin, which generates apoptotic bodies [11,12,13,14]. The mitochondrial pathway of apoptosis, regulated by the *BCL-2* family, is induced when its anti-apoptotic components are neutralized by *BH3*, *BAK* and *BAX* proteins, leading to the rupture of the outer mitochondrial membrane and inducing its permeabilization, ultimately inducing the activation of initiator caspase 9 and effector caspases 3 and 7 [15,16,17,18,19]. In our study, we observed that the fungicide ipconazole had the ability to increase caspase 3/7 enzyme activity and to increase the molecular expression of biomarkers related to cell death activation: *BAX*, *BNIP3*, *APAF1*, *AKT1* and *CASP3*. Other in vitro and in vivo studies confirmed the ability of fungicides to increase the activity of caspase enzymes. Epoxiconazole was able to induce caspase 3 activity in F98 glioma cells at a concentration of 12–50 µM [25,34], in PC12 cells at a concentration of 5-20 µM [33] and in neural stem cells at a concentration of 2–10 µM [24]; in three human cell lines (*HepG2*, *HEK293* and *JEG3*), caspase 3/7 was induced at a concentration of 125 g/L [41]. Tebuconazole increased caspase 9 and caspase 3 activation in myocardial cells at doses of 0.9, 9 and 27 mg/kg b. w. [42]; in bovine mammary gland epithelial cells at 100–200 µM [43]; in HCT116 cells at increasing doses of 12. 5, 25, 50 and 75 µM [40]; and in cardiac cells at concentrations of 30 and 60 µM [10]. It also increased caspase 3/7 activity in three human cell lines (*HepG2*, *HEK293* and *JEG3*) at a concentration of 50 ppm [41]; in rat kidney cells, caspase 3/7 was induced at 175–200 µg/L [44]; and propiconazole in zebrafish increased caspase 3/7 activity by 1 µg/L [45]. Likewise, studies such as ours also found that triazole fungicides are able to alter the gene expression of te cell death biomarkers *BAX*, *BCL2*, *BNIP3*, *APAF1* and *AKT1* [37,43,46,47,48,49].

The *NLRP3* inflammasome is a multimeric protein complex that assembles in the cytosol and serves as a scaffold to recruit the inactive zymogen pro-caspase 1, which is activated to caspase 1 after oligomerization. Caspase 1 is an enzyme that cleaves the precursor cytokines pro-IL1β and pro-IL18 to their active biological forms, *IL1β* and *IL18*, and can also induce pyroptosis [50]. Furthermore, *IL-1β* could induce *TNFα* expression and *IL6* secretion, which may trigger an increased cellular inflammatory response [51,52]. In contrast, it was proposed that *NFκB*, the master inflammatory regulator, which is not part of the inflammasome complex, could increase the gene production of NLRP3 and its proinflammatory mediators [53]. In our study, we determined that inflammasome complex components (*NLRP3*, *CASP1* and *IL1β*) and proinflammatory biomarkers (*NFκB*, *IL6* and *TNFα*) were up-regulated in EA.hy926 cells exposed to ipconazole, with a significant association between them. Several in vitro studies with pesticides showed that the *NLRP3* inflammasome complex was overexpressed and that inflammatory components showed significant increases after exposure to pesticides such as rotenone, pyrethroids, paraquat and chlorpyrifos [18,19,54,55,56,57]. This study provided some of the possible cellular events related to cell death, oxidative stress and inflammation that may be altered in EA.hy926 cells as a result of ipconazole exposure, which was complemented by the results of a study recently published in the same journal on the effect of ipconazole on SH-SY5Y cells [58].

## 5. Conclusions

The present study demonstrates, in EA.hy926 endothelial cells exposed to the fungicide ipconazole, a dose-dependent reduction in cell viability, and the results are confirmed by the increase in caspase 3/7 enzyme activity and the molecular expression of the cell death biomarkers *BAX*, *APAF1*, *BNIP3*, *CASP3* and *AKT1*. We also propose that this cytotoxic effect could be due to the fact that ipconazole induced oxidative stress by increasing ROS generation and decreasing the molecular expression of *NRF2* and *GPx* as well as the induction of the proinflammatory mediators *NLRP3*, *CASP1*, *IL1β*, *NFκB*, *IL6* and *TNFα*. Due to the widespread use and abuse of this chemical compound, further studies in various cells and at the systemic level in animals are needed to better understand the cytotoxic effects and their implication in the deterioration of human, animal and environmental health.

## Figures and Tables

**Table 1:** Forward and reverse sequences for genes related to cell death, the inflammasome complex and antioxidant biomarkers.

Gene	Forward	Reverse
*Cell Death*		
*BAX* (BCL-2-associated X protein)	CCCCCGAGAGGTCTTTTTCC′	CCTTGAGCACCAGTTTGCTG′
*CASP3* (caspase 3)	‘GTGGAGGCCGACTTCTTGTA′	TTTCAGCATGGCACAAAGCG′
*APAF1* (apoptotic protease-activating factor 1)	TCTTCCAGTGGTAAAGATTCAGTT′	CGGAGACGGTCTTTAGCCTC′
*BNIP3* (BCL2-interacting protein 3)	CCTCAGCATGAGGAACACGA′	GCCACCCCAGGATCTAACAG′
*BCL2* (B-cell lymphoma 2)	′TCTCATGCCAAGGGGGAAAC′	TCCCGGTTATCGTACCCTGT′
*P53* (tumor protein P53)	GAACAAGTTGGCCTGCACTG	GAAGTGGGCCCCTACCTAGA
*AKT1* (AKT serine/threonine kinase 1)	GAAGGACGGGAGCAGGC	TGTACTCCCCTCGTTTGTGC
*Inflammasome Complex*		
*NLRP3* (NLR pyrin domain containing 3)	CCCCGTAATCAACGGGACAA′	AGCCAAATGCTTACCAGAAAGT′
*CASP1* (caspase 1)	′GAAAAGCCATGGCCGACAAG′	GCCCCTTTCGGAATAACGGA′
*IL1β* (interleukin-1 beta)	′CCAGCTACGAATCTCCGACC′	TATCCTGTCCCTGGAGGTGG
*NFκB* (nuclear factor kappa B)	′TTTTCGACTACGCGGTGACA′	GTTACCCAAGCGGTCCAGAA′
*TNFα* (tumor necrosis factor alpha)	CTGGAAAGGACACCATGAGCA′	TCTCTCAGCTCCACGCCATT′
*IL6* (interleukin 6)	‘CCAGTACCCCCAGGAGAAGA′	CAGCTCTGGCTTGTTCCTCA′
*Antioxidant Biomarkers*		
*NRF2* (nuclear factor erythroid related factor2)	CTGGTCATCGGAAAACCCCA′	TCTGCAATTCTGAGCAGCCA’
*SOD* (superoxide dismutase)	′CCACTGCTGGGGATTGATGT′	CGTGGTTTACTTTTTGCAAGCC′
*GPx* (glutathione peroxidase)	′TTCGAGCCCAACTTCATGCT′	′CGATGTCAGGCTCGATGTCA′
*Normalizer*		
*GAPDH* (glyceraldehyde-3-phosphate dehydrogenase)	′GAGAAGGCTGGGGCTCATTT	AGTGATGGCATGGACTGTGG′

**Table 2:** Gene expression of cell-death-related molecular biomarkers in EA.hy926 cells treated with ipconazole at 20 and 50 µM or DMSO (0.1%) as a carrier control.

Gene	Control	Vehicle (DMSO)	Ipconazole	
			20 µM	50 µM
			Fold Change	
*BAX*	1.00	0.99	1.39 **	1.73 ***
*BCL2*	1.00	0.97	0.13 ***	0.16 ***
*APAF1*	1.00	0.99	1.35	2.66 ***
*BNIP3*	1.00	1.02	1.08	2.08 ***
*CASP3*	1.00	1.00	1.66 *	2.24 ***
*AKT1*	1.00	1.02	1.27	2.42 ***
*P53*	1.00	0.92	0.95	1.04

Data for gene expression represent fold change mean. * *p* < 0.05, ** *p* < 0.01 or *** *p* < 0.001 compared to vehicle, for three independent experiments. The red letter represents up-regulation, and the green letter represents down-regulation.

**Table 3:** Gene expression of inflammasome-related molecular biomarkers in EA.hy926 cells treated with ipconazole at 20 and 50 µM or DMSO (0.1%) as a carrier control.

Gene	Control	Vehicle (Veh)	Ipconazole	
			20 µM	50 µM
			Fold Change	
*NLRP3*	1.00	0.97	1.52	1.59 *
*CASP1*	1.00	1.01	0.96	2.07 ***
*IL1β*	1.00	0.97	2.14 **	2.41 **
*NFκB*	1.00	1.02	1.76	2.31 **
*IL6*	1.00	0.96	1.37	3.34 ***
*TNFα*	1.00	1.00	1.30	1.56 *

Data for gene expression represent fold change mean. * *p* < 0.05, ** *p* < 0.01 or *** *p* < 0.001 compared to vehicle, for three independent experiments. The red color represents up-regulation.

**Table 4:** Gene expression of antioxidant-related molecular biomarkers in EA.hy926 cells treated with ipconazole at 20 and 50 µM or DMSO (0.1%) as a carrier control.

Gene	Control	Vehicle (Veh)	Ipconazole	
			20 µM	50 µM
			Fold Change	
*NRF2*	1.00	0.98	0.98	0.56 **
*SOD*	1.00	1.06	0.92	0.90
*GPx*	1.00	0.97	0.97	0.45 ***

Data for gene expression represent fold change mean. ** *p* < 0.01 or *** *p* < 0.001 compared to vehicle, for three independent experiments. The green color represents down-regulation.

## Data Availability

Not applicable.
